# Supplementary material for: Defining priority areas for conservation of poorly known species: A case study of the endemic Brazilian three-banded armadillo
Source: Camb Prism Extinct. 2023 Jan 5;1:e2. doi: 10.1017/ext.2022.2 (PMC11895736; doi:10.1017/ext.2022.2)
Supplement: Supplementary file 1 [file S275509582200002Xsup.zip › S275509582200002Xsup002.pdf]

Supplementary Information for

Defining priority areas for conservation of poorly known species: a case study of the endemic Brazilian three-banded armadillo

Anderson Feijó[*], Rodolfo Assis Magalhães, Adriana Bocchiglieri, José Luís P. Cordeiro, Liana Mara Mendes de Sena, Nina Attias

*Corresponding author: andefeijo@gmail.com

This PDF file includes:

Supplementary text
Figures S1 to S4
Table S1 to S6
Legend for Dataset S1
Supplementary references

Other supplementary materials for this manuscript include the following:

Dataset S1

Supplementary Text

Species distribution modeling performance. All five modeling algorithms had high Area Under the Curve (ranged from 0.952 to 0.988; Table S6), high TSS (ranged from 0.822 to 0.943), and low omission rates (0 - 0.04), indicating low overfit and high performance in discriminating occurrences from background points (Phillips et al., 2006). The SDM output of each model yielded overall similar results, showing the central portion of Caatinga and the northeastern portion of Cerrado as the highest suitability areas for the three-banded armadillo, while peripheral areas close to the Atlantic Forest on the east and to Amazon on the west and north are deemed as unsuitable or of very low suitability (Supplementary Fig. S4). Uplands are clearly differentiated from lowlands with greater suitability values in Maxent and boosted regression tree models, whereas in the other three models they are less differentiated. Nevertheless, areas with high habitat suitability in the ensemble output accord with the species distribution limits previously proposed by Feijó et al. (2015). Therefore, we consider our SDM models to provide informative habitat predictions of the Brazilian three-banded armadillo.

Supplementary Figures

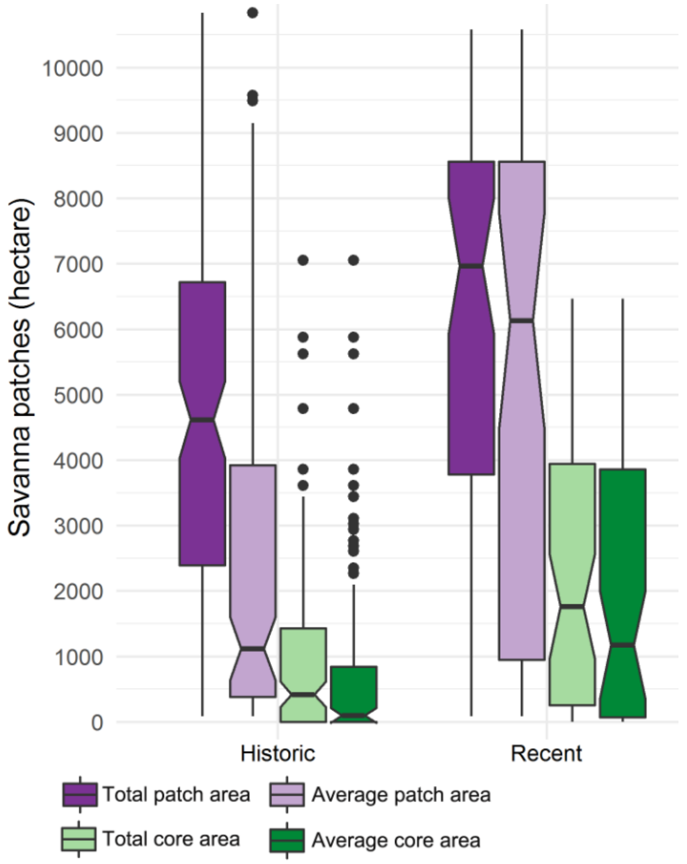

Figure S1. Boxplot comparing the extent of savanna patches surrounding (squared 5 km buffer) historical (before 2000) and recent (since 2000) records of Brazilian three-banded armadillo. Note that recent records are located in areas with a greater amount of savanna remnants.

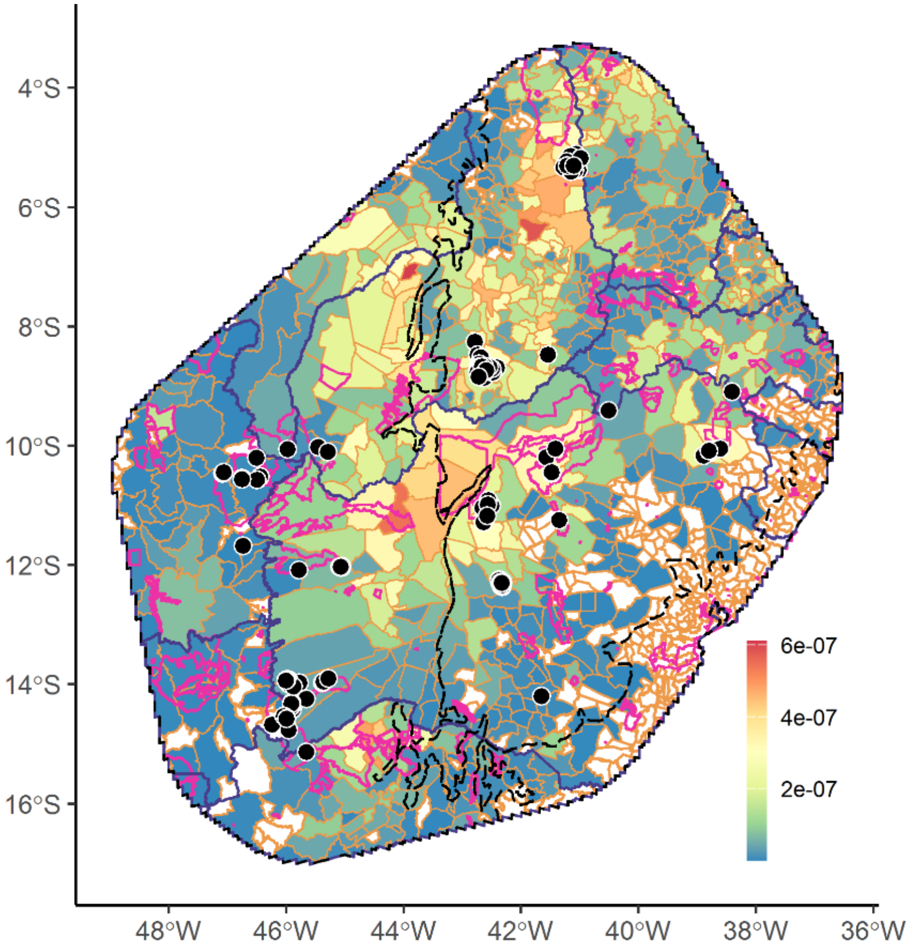

Figure S2. Relative proportion of savanna core areas across Brazilian municipalities. Warmer colors represent municipalities with a higher amount of core cells in relation to the municipality total area, indicating municipalities that would benefit from proactive conservation management strategies. White polygons indicate municipalities with no core area remnant. For a complete list see Dataset S1. Black dots represent recent records of Brazilian three-banded armadillos. Black lines delimit the Cerrado and Caatinga, blue lines delimit the Brazilian states, and orange lines delimit the municipalities. The region displayed comprehends the 200-km convex hull from the marginal recent records.

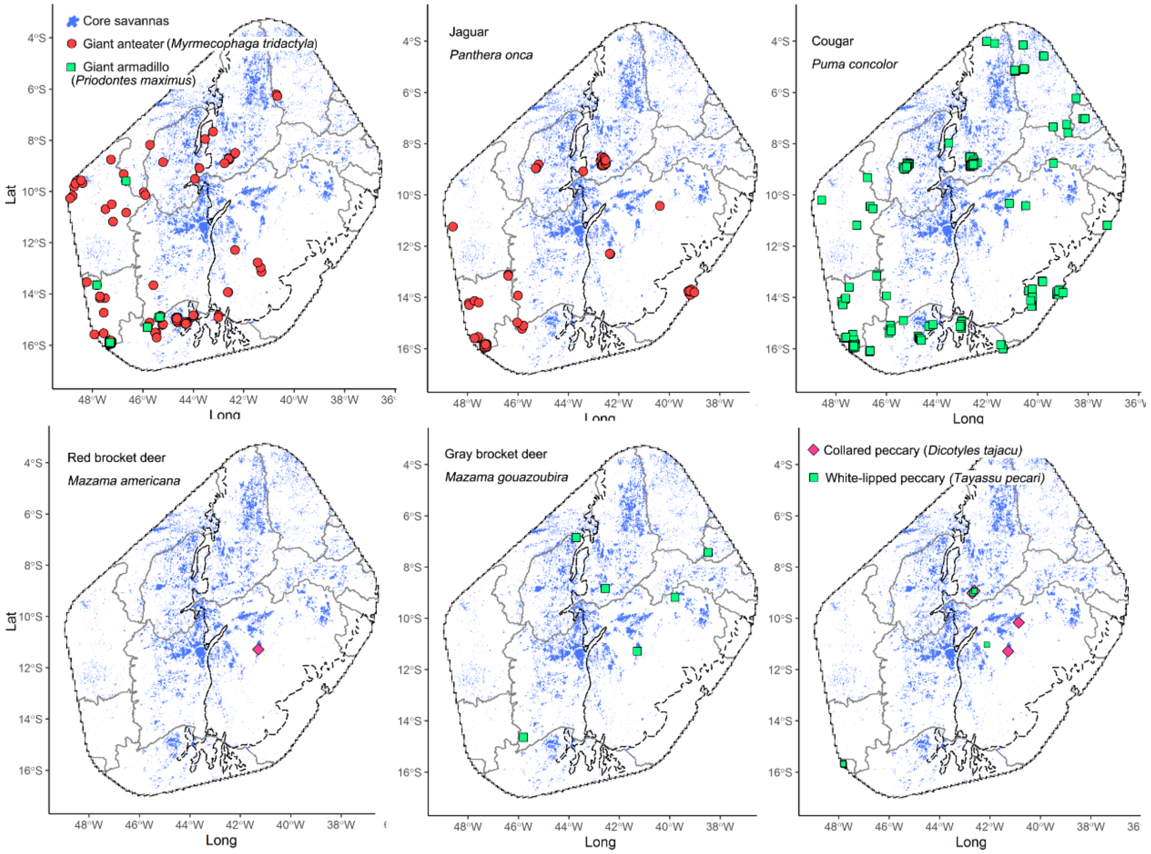

Figure S3. Records of threatened mammal species within the distribution range of the Brazilian three-banded armadillo (*Tolypeutes tricinctus*). Core savanna areas are shown in blue. Data for giant anteater (*Myrmecophaga tridactyla*) and giant armadillo (*Priodontes maximus*) obtained from Santos et al. (2019), for jaguar (*Panthera onca*) and cougar (*Puma concolor*) from Nagy-Reis et al. (2019), and for red brocket deer (*Mazama americana*), gray brocket deer (*Mazama gouazoubira*), collared peccary (*Dicotyles tajacu*), and white-lipped peccary (*Tayassu pecari*) from Global Biodiversity Information Facility (GBIF, accessed on 9 September 2022; https://doi.org/10.15468/dl.adv3cp). When available, we only included records since the year 2000, representing recent populations. Black lines delimit the biomes and gray lines delimit the Brazilian states. Note that the recent records of these species in the Caatinga are mainly restricted to the core savanna areas in the western portion of the biome, overlapping with the remnant three-banded armadillo populations.

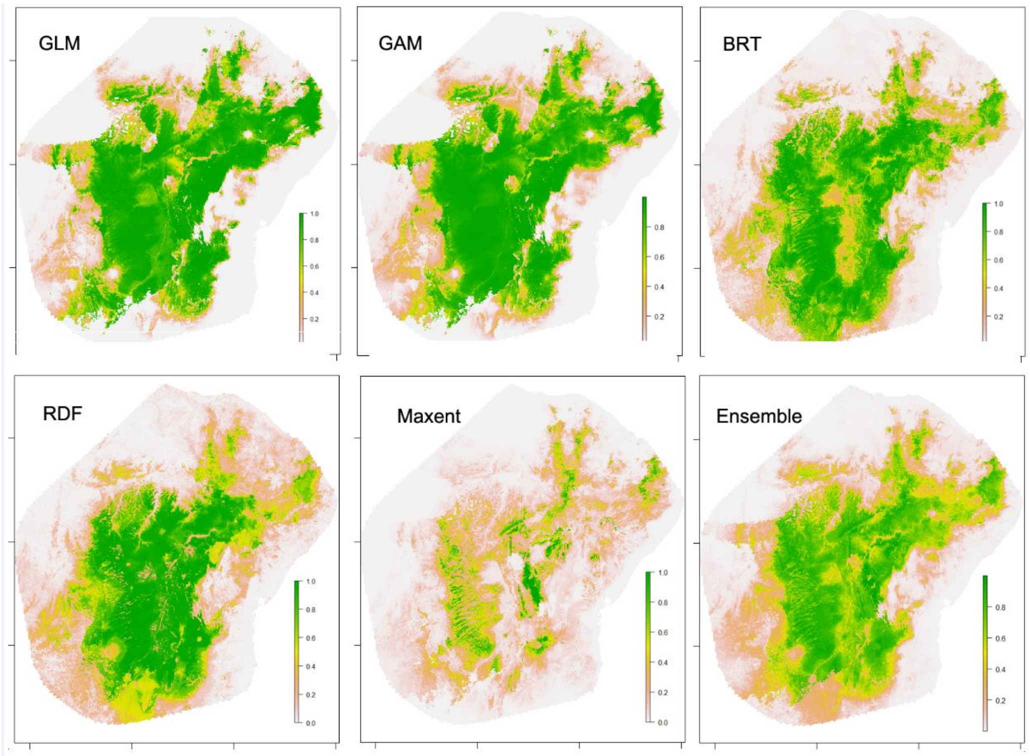

Figure S4. Probabilistic species distribution models of the five algorithms and the ensemble model for the Brazilian three-banded armadillo. Ensemble output based on the average of suitability values weighted by the algorithm-specific TSS performance. GLM, generalized linear models; GAM, generalized additive models; BRT, boosted regression trees; RDF, random forest.

Supplementary Tables

Table S1. Recent localities of the Brazilian three-banded armadillo (*Tolypeutes tricinctus*) used in this study.

Longitude	Latitude	Locality	Brazilian State	Year	Source
-45.0709	-12.0427	Barreiras	Bahia	2019	Field record - This study
-45.0809	-12.0349	Barreiras	Bahia	2019	Field record - This study
-45.0795	-12.0346	Barreiras	Bahia	2019	Field record - This study
-45.0454	-12.0408	Barreiras	Bahia	2020	Field record - This study
-45.0521	-12.0251	Barreiras	Bahia	2020	Field record - This study
-45.0629	-12.0354	Barreiras	Bahia	2019	Field record - This study
-42.3627	-12.2908	Brotas de Macaubas	Bahia	2017	Magalhaes et al. (2021)
-42.367	-12.2539	Brotas de Macaubas, Comunidade Sumidouro	Bahia	2019	Field record - This study
-42.3367	-12.2855	Brotas de Macaubas, Comunidade Sumidouro	Bahia	2019	Field record - This study
-42.318	-12.3146	Brotas de Macaubas, Comunidade Sumidouro	Bahia	2019	Field record - This study
-42.3552	-12.2634	Brotas de Macaubas, Comunidade Sumidouro	Bahia	2019	Field record - This study
-42.3037	-12.2976	Brotas de Macaubas, Comunidade Sumidouro	Bahia	2019	Field record - This study
-42.3555	-12.3041	Brotas de Macaubas, Comunidade Sumidouro	Bahia	2019	Field record - This study
-42.3337	-12.3221	Brotas de Macaubas, Comunidade Sumidouro	Bahia	2019	Field record - This study
-42.3042	-12.312	Brotas de Macaubas, Comunidade Sumidouro	Bahia	2019	Field record - This study
-42.3359	-12.3556	Brotas de Macaubas, Comunidade Sumidouro	Bahia	2019	Field record - This study
-42.3154	-12.2914	Brotas de Macaubas, Comunidade Sumidouro	Bahia	2019	Field record - This study
-42.3554	-12.3041	Brotas de Macaubas, Comunidade Sumidouro	Bahia	2019	Field record - This study
-42.3552	-12.2635	Brotas de Macaubas, Comunidade Sumidouro	Bahia	2019	Field record - This study
-42.3042	-12.3118	Brotas de Macaubas, Comunidade Sumidouro	Bahia	2019	Field record - This study
-42.3154	-12.2915	Brotas de Macaubas, Comunidade Sumidouro	Bahia	2020	Field record - This study
-42.3337	-12.3221	Brotas de Macaubas, Comunidade Sumidouro	Bahia	2020	Field record - This study
-42.3178	-12.302	Brotas de Macaubas, Comunidade Sumidouro	Bahia	2017	Field record - This study
-42.3372	-12.3389	Brotas de Macaubas, Comunidade Sumidouro	Bahia	2017	Field record - This study
-42.3174	-12.3364	Brotas de Macaubas, Comunidade Sumidouro	Bahia	2017	Field record - This study
-42.3152	-12.3026	Brotas de Macaubas, Comunidade Sumidouro	Bahia	2019	Field record - This study
-42.3442	-12.2942	Brotas de Macaubas, Comunidade Sumidouro	Bahia	2019	Field record - This study
-42.315	-12.3712	Brotas de Macaubas, Comunidade Sumidouro	Bahia	2019	Field record - This study

-42.3611	-12.298	Brotas de Macaubas, Comunidade Sumidouro	Bahia	2019	Field record - This study
-42.3265	-12.3355	Brotas de Macaubas, Comunidade Sumidouro	Bahia	2019	Field record - This study
-42.3436	-12.2847	Brotas de Macaubas, Comunidade Sumidouro	Bahia	2019	Field record - This study
-42.3393	-12.2864	Brotas de Macaubas, Comunidade Sumidouro	Bahia	2019	Field record - This study
-42.3206	-12.3032	Brotas de Macaubas, Comunidade Sumidouro	Bahia	2019	Field record - This study
-42.3186	-12.3026	Brotas de Macaubas, Comunidade Sumidouro	Bahia	2019	Field record - This study
-42.3206	-12.3029	Brotas de Macaubas, Comunidade Sumidouro	Bahia	2019	Field record - This study
-42.3242	-12.3034	Brotas de Macaubas, Comunidade Sumidouro	Bahia	2019	Field record - This study
-42.3257	-12.3357	Brotas de Macaubas, Comunidade Sumidouro	Bahia	2019	Field record - This study
-42.3369	-12.2966	Brotas de Macaubas, Comunidade Sumidouro	Bahia	2019	Field record - This study
-42.3201	-12.3003	Brotas de Macaubas, Comunidade Sumidouro	Bahia	2019	Field record - This study
-42.3193	-12.3008	Brotas de Macaubas, Comunidade Sumidouro	Bahia	2019	Field record - This study
-42.3448	-12.2947	Brotas de Macaubas, Comunidade Sumidouro	Bahia	2019	Field record - This study
-42.3149	-12.3369	Brotas de Macaubas, Comunidade Sumidouro	Bahia	2019	Field record - This study
-42.3171	-12.3039	Brotas de Macaubas, Comunidade Sumidouro	Bahia	2019	Field record - This study
-42.3178	-12.3044	Brotas de Macaubas, Comunidade Sumidouro	Bahia	2019	Field record - This study
-42.3211	-12.3031	Brotas de Macaubas, Comunidade Sumidouro	Bahia	2019	Field record - This study
-42.3732	-12.257	Brotas de Macaubas, Comunidade Sumidouro	Bahia	2020	Field record - This study
-42.3205	-12.3003	Brotas de Macaubas, Comunidade Sumidouro	Bahia	2020	Field record - This study
-42.3249	-12.3028	Brotas de Macaubas, Comunidade Sumidouro	Bahia	2020	Field record - This study
-42.326	-12.3025	Brotas de Macaubas, Comunidade Sumidouro	Bahia	2020	Field record - This study
-42.3444	-12.2956	Brotas de Macaubas, Comunidade Sumidouro	Bahia	2020	Field record - This study
-42.3198	-12.3032	Brotas de Macaubas, Comunidade Sumidouro	Bahia	2020	Field record - This study
-41.65	-14.2	Brumado	Bahia	2001	Freitas and Silva (2005)
-38.8832	-10.1664	Canudos, Estacao Eolica Canudos I	Bahia	2018	Field record - This study
-38.7982	-10.1283	Canudos, Estacao Eolica Canudos II and III	Bahia	2021	Field record - This study
-38.7879	-10.0874	Canudos, Estacao Eolica Canudos II_III	Bahia	2021	Field record - This study
-38.7935	-10.0874	Canudos, Estacao Eolica Canudos III	Bahia	2021	Field record - This study
-42.6558	-11.0475	Gentio do Ouro	Bahia	2021	Field record - This study
-42.6564	-11.0475	Gentio do Ouro	Bahia	2021	Field record - This study
-42.5434	-11.0178	Gentio do Ouro	Bahia	2022	Field record - This study
-42.4948	-11.0078	Gentio do Ouro	Bahia	2021	Field record - This study
-42.6364	-11.284	Gentio do Ouro, Gameleira do Assurua	Bahia	2022	Field record - This study

-42.5667	-11.1811	Gentio do Ouro, Gameleira do Assurua	Bahia	2021	Field record - This study
-42.5611	-10.9868	Gentio do Ouro, Santo Inacio	Bahia	2021	Field record - This study
-38.4	-9.1	Gloria	Bahia	2012	Feijo et al. (2015)
-42.5505	-10.9159	Itaguacu	Bahia	2021	Field record - This study
-45.9093	-14.3708	Jaborandi, APA Nascentes do Rio Vermelho	Bahia	2021	Field record - This study
-45.9089	-14.3321	Jaborandi, APA Nascentes do Rio Vermelho	Bahia	2021	Field record - This study
-46	-13.9333	Jaborandi, Jatoba Farm	Bahia	2009	Bocchiglieri et al. (2012)
-45.9517	-14.7738	Jaborandi, Jatoba Farm	Bahia	2021	Field record - This study
-45.9844	-14.5714	Jaborandi, Jatoba Farm	Bahia	2021	Field record - This study
-45.9679	-13.9383	Jaborandi, Jatoba Farm	Bahia	2008	Field record - This study
-45.9509	-13.9966	Jaborandi, Jatoba Farm	Bahia	2008	Field record - This study
-45.7669	-13.9788	Jaborandi, Jatoba Farm	Bahia	2008	Field record - This study
-45.9617	-13.9206	Jaborandi, Jatoba Farm	Bahia	2008	Field record - This study
-45.9506	-13.9466	Jaborandi, Jatoba Farm	Bahia	2008	Field record - This study
-45.9466	-13.9443	Jaborandi, Jatoba Farm	Bahia	2008	Field record - This study
-45.8697	-14.0379	Jaborandi, Jatoba Farm	Bahia	2008	Field record - This study
-45.9793	-13.928	Jaborandi, Jatoba Farm	Bahia	2009	Field record - This study
-46.0027	-13.9386	Jaborandi, Jatoba Farm	Bahia	2009	Field record - This study
-45.8478	-14.2581	Jaborandi, Refugio da Visa Silvestre Veredas do Oeste Baiano	Bahia	2021	Field record - This study
-45.8896	-14.4235	Jaborandi, Refugio da Visa Silvestre Veredas do Oeste Baiano	Bahia	2021	Field record - This study
-45.3744	-13.9751	Jaborandi, Refugio da Visa Silvestre Veredas do Oeste Baiano	Bahia	2021	Field record - This study
-45.3655	-13.9651	Jaborandi, Refugio da Visa Silvestre Veredas do Oeste Baiano	Bahia	2021	Field record - This study
-45.3633	-13.9584	Jaborandi, Refugio da Visa Silvestre Veredas do Oeste Baiano	Bahia	2021	Field record - This study
-45.276	-13.9023	Jaborandi, Refugio da Visa Silvestre Veredas do Oeste Baiano	Bahia	2021	Field record - This study
-45.6537	-14.2498	Jaborandi, Refugio da Visa Silvestre Veredas do Oeste Baiano	Bahia	2021	Field record - This study
-45.8979	-14.3099	Jaborandi, Refugio da Visa Silvestre Veredas do Oeste Baiano	Bahia	2021	Field record - This study
-38.7478	-10.0704	Jeremoabo, Estacao Eolica Canudos II and III	Bahia	2021	Field record - This study
-38.6065	-10.056	Jeremoabo, Estacao Eolica Canudos IV	Bahia	2021	Field record - This study
-40.5035	-9.4168	Juazeiro	Bahia	2015	Bioface-Public Database
-45.7805	-12.0905	Luis Eduardo Magalhes	Bahia	2017	Bioface-Public Database
-41.35	-11.25	Morro do Chapeu, Chapada Diamantina, Lages	Bahia	2004	Feijo et al. (2015)
-41.5667	-10.2	Sento Se	Bahia	2012	Feijo et al. (2015)
-41.406	-10.0573	Sento Se	Bahia	2020	Field record - This study
-41.4695	-10.4695	Umburanas	Bahia	2019	Field record - This study
-41.4782	-10.4512	Umburanas, APA Boqueirao da Onca	Bahia	2020	Field record - This study
-42.6404	-11.0578	Xique-xique	Bahia	2016	Feijo et al. (2015)
-46.2397	-14.6802	Mambai	Goias	2013	Hannibal et al. (2021)
-46.0375	-14.5349	Mambai	Goias	2021	Field record - This study

-45.99	-14.5849	Mambai	Goias	2021	Field record - This study
-45.9679	-10.0651	Nascentes do Rio Parnaiba National Park	Maranhao	2016	Bioface-Public Database
-45.6494	-15.1414	Chapada Gaucha	Minas Gerais	2014	Schetino et al. (2021)
-42.7833	-8.26667	Brejo do Piaui	Piaui	2010	Feijo et al. (2015)
-41.0977	-5.31177	Buriti dos montes	Piaui	2020	Field record - This study
-41.0956	-5.3136	Buriti dos montes	Piaui	2019	Bioface-Public Database
-41.0608	-5.11422	Buriti dos Montes, Bebedouro	Piaui	2020	Field record - This study
-41.1906	-5.2808	Buriti dos Montes, Cacimb?o	Piaui	2020	Field record - This study
-41.1568	-5.15116	Buriti dos Montes, Cana Brava	Piaui	2020	Field record - This study
-41.2217	-5.23598	Buriti dos Montes, Dois Morros	Piaui	2020	Field record - This study
-41.2087	-5.36376	Buriti dos Montes, Esquisito	Piaui	2020	Field record - This study
-41.0167	-5.38624	Buriti dos Montes, Janela	Piaui	2020	Field record - This study
-40.9793	-5.18111	Buriti dos Montes, Jatoba Medonho	Piaui	2020	Field record - This study
-41.0771	-5.39313	Buriti dos Montes, Morro do Jati	Piaui	2020	Field record - This study
-41.2708	-5.33661	Buriti dos Montes, Palmas	Piaui	2020	Field record - This study
-41.1455	-5.41092	Buriti dos Montes, Peba	Piaui	2020	Field record - This study
-41.2124	-5.3134	Buriti dos Montes, Tranqueira	Piaui	2020	Field record - This study
-45.2867	-10.1063	Gilbues	Piaui	2019	Field record - This study
-41.5346	-8.47715	Lagoa do Barro do Piaui	Piaui	2020	Bioface-Public Database
-45.4517	-10.0275	Sao Goncalo do Gurgueia	Piaui	2019	inaturalist-Public Database
-42.713	-8.63697	Serra da Capivara National Park	Piaui	2017	Magalhaes et al. (2021)
-42.7301	-8.82082	Serra da Capivara National Park	Piaui	2018	Field record - This study
-42.6831	-8.8586	Serra da Capivara National Park	Piaui	2018	Field record - This study
-42.7362	-8.47576	Serra da Capivara National Park	Piaui	2018	Field record - This study
-42.7269	-8.6473	Serra da Capivara National Park	Piaui	2018	Field record - This study
-42.63	-8.86863	Serra da Capivara National Park	Piaui	2018	Field record - This study
-42.407	-8.69861	Serra da Capivara National Park	Piaui	2018	Field record - This study
-42.4781	-8.77391	Serra da Capivara National Park	Piaui	2018	Field record - This study
-42.6762	-8.521	Serra da Capivara National Park	Piaui	2018	Field record - This study
-42.7038	-8.62913	Serra da Capivara National Park	Piaui	2018	Field record - This study
-42.7296	-8.68322	Serra da Capivara National Park	Piaui	2018	Field record - This study
-42.7239	-8.69823	Serra da Capivara National Park	Piaui	2018	Field record - This study
-42.7348	-8.73308	Serra da Capivara National Park	Piaui	2018	Field record - This study
-42.7356	-8.75802	Serra da Capivara National Park	Piaui	2018	Field record - This study
-42.7355	-8.77271	Serra da Capivara National Park	Piaui	2018	Field record - This study
-42.4912	-8.71651	Serra da Capivara National Park	Piaui	2018	Field record - This study
-42.5355	-8.6901	Serra da Capivara National Park	Piaui	2018	Field record - This study
-42.6131	-8.67239	Serra da Capivara National Park	Piaui	2018	Field record - This study
-42.6659	-8.67672	Serra da Capivara National Park	Piaui	2018	Field record - This study
-42.606	-8.76671	Serra da Capivara National Park	Piaui	2018	Field record - This study
-42.5889	-8.7356	Serra da Capivara National Park	Piaui	2018	Field record - This study
-42.7185	-8.84564	Serra da Capivara National Park	Piaui	2018	Field record - This study
-42.6667	-8.58333	Serra das Confusoes National Park, Canto Verde	Piaui	2002	Feijo et al. (2015)
-47.0748	-10.47	Alto_da_Muricoca,NA	Tocatins	2014	Field record - This study

-46.7333	-11.6833	Dianopolis	Tocatins	2010	Feijo et al. (2015)
-47.0644	-10.4502	ESEC Estrada Borda	Tocatins	2014	Field record - This study
-46.4958	-10.5797	Estrada,NA	Tocatins	2012	Field record - This study
-46.4339	-10.5203	Parque Estadual do Jalapao, Mateiros	Tocatins	2020	Field record - This study
-46.7531	-10.5531	Parque Estadual do Jalapao, Rio Novo	Tocatins	2020	Field record - This study
-46.5057	-10.21	Parque Estadual do Jalapao, Sao Felix do Tocantins	Tocatins	2020	Field record - This study
-46.7471	-10.5681	TO-255	Tocatins	2013	Field record - This study
-46.4921	-10.5789	TO-255_próximo_Mateiros	Tocatins	2012	Field record - This study

Table S2. Reclassification of land-cover classes. Original land-cover classification based on MapBiomas Collection 6. Names in red represent new classification.

Original Classification	Reclassification
Forest	Forest
Savanna	Savanna
Mangrove	Mangrove
Forest Plantation	Forest Plantation
Wetlands	Wetlands
Grassland	Grassland
non-Forest Formations	non-Forest Formations
Pasture	Pasture
Sugar cane	Agriculture
Mosaic Agriculture and Pasture	Mosaic Agriculture and Pasture
Beach Dune and Sand Spot	Dune
Urban Area	Urban Area
Other non-Vegetated Areas	Other non-Vegetated Areas
Rocky Outcrop	Rocky Outcrop
Mining	Mining
Aquaculture	Water
Salt Flat	Mangrove
River, Lake, and Ocean	Water
Soybean	Agriculture
Rice	Agriculture
Temporary Crops	Agriculture
Coffee	Agriculture
Other Perennial Crop	Agriculture
Wooded Restinga	Mangrove

Table S3. Proportion of land-cover types across the Brazilian three-banded armadillo habitat suitability categories.

Land Cover	Habitat suitability categories						Total (%)
	Unsuitable	Very Low	Low	Moderate	High	Very High	
Savanna	1.63	6.13	8.27	9.89	10.21	5.59	41.72
Pasture	2.69	5.38	4.95	3.96	3.64	1.24	21.86
Forest	3.43	4.47	2.29	1.52	0.78	0.34	12.83
Agriculture & Pasture	0.77	1.59	1.63	1.78	2.03	0.71	8.51
Grassland	0.19	0.57	1.42	1.52	1.89	1.63	7.22
Agriculture	0.23	0.83	1.07	0.81	1.28	0.32	4.54
Wetlands	0.1	0.49	0.06	0.06	0.06	0.07	0.84
Water	0.13	0.19	0.17	0.15	0.05	0.01	0.7
Forest Plantation	0.05	0.06	0.16	0.29	0.07	0.02	0.65
Urban Area	0.1	0.19	0.12	0.06	0.05	0.01	0.53
Other Non-vegetaded Area	0.02	0.06	0.07	0.06	0.07	0.04	0.32
Rocky Outcrop	0	0.02	0.03	0.02	0.02	0.01	0.1
Dune	0.05	0.03	0	0.01	0.01	0	0.1
Mangrove	0.07	0.02	0	0	0	0	0.09

Table S4. Proportion of the Brazilian three-banded armadillo habitat suitability categories within protected areas.

Environmental Suitability Categories	Within PAs (%)	
	No	Yes
Unsuitable	85.68	14.32
Very Low	89.23	10.77
Low	91.44	8.56
Moderate	91.88	8.12
High	89.65	10.35
Very High	78.46	21.54

Table S5. Results of t-test comparing savanna patches surrounding (squared 5 km buffer) historical and recent records of the Brazilian three-banded armadillo. P.A., patch area, C.A., core area, CI, confidence interval. Patch metric values in hectares.

Savanna metrics	t value	df	*p*-value	Mean (historical)	Mean (recent)	95% CI (historical)	95% CI (recent)
Total P.A	4.6202	101.77	1.13E-05	3659.04	6195.024	3624.734	1447.234
Average P.A.	4.8486	95.852	4.80E-06	1894.766	5063.089	4465.446	1871.199
Total C.A.	4.0116	95.016	0.00012	856.944	2303.136	2161.8773	730.5067
Average C.A.	4.0264	92.551	0.000116	656.348	2127.753	2197.1352	745.6753

Table S6. Evaluation output for the five niche model algorithms and three different thresholds. Algorithms: BRT, Boosted regression trees; GAM, Generalized additive models; GLM, Generalized linear models; MXD, Maxent; RDF, Random Forest. Ensemble method: WMEA, Weighted mean.Threshold for binary maps: LPT, Least Presence Threshold, MAX_TSS, True Skill Statistics.

Algorithm	Threshold	AUC	Kappa	TSS	Jaccard	Sorensen	AUC_SD	Kappa_SD	TSS_SD	Jaccard_SD	Sorensen_SD
BRT	JACCARD	0.986022	0.919333	0.919333	0.925249	0.96104	0.016947	0.028324	0.028324	0.024467	0.013168
BRT	LPT	0.986022	0.886	0.886	0.901249	0.947151	0.016947	0.081117	0.081117	0.061592	0.035098
BRT	MAX_TSS	0.986022	0.919333	0.919333	0.925249	0.96104	0.016947	0.028324	0.028324	0.024467	0.013168
GAM	JACCARD	0.976102	0.895333	0.895333	0.906108	0.949831	0.028866	0.077732	0.077732	0.061655	0.035216
GAM	LPT	0.976102	0.838	0.838	0.866157	0.926788	0.028866	0.104019	0.104019	0.077898	0.044659
GAM	MAX_TSS	0.976102	0.895333	0.895333	0.906108	0.949831	0.028866	0.077732	0.077732	0.061655	0.035216
GLM	JACCARD	0.986744	0.927667	0.927667	0.932568	0.964055	0.01531	0.076772	0.076772	0.067833	0.037486
GLM	LPT	0.986744	0.822333	0.822333	0.858468	0.921292	0.01531	0.134885	0.134885	0.102075	0.058119
GLM	MAX_TSS	0.986744	0.927667	0.927667	0.932568	0.964055	0.01531	0.076772	0.076772	0.067833	0.037486
MXD	JACCARD	0.952661	0.839	0.839	0.861936	0.925293	0.03935	0.068593	0.068593	0.046656	0.02773
MXD	LPT	0.952661	0.831	0.831	0.857427	0.922724	0.03935	0.064846	0.064846	0.044975	0.026746
MXD	MAX_TSS	0.952661	0.839	0.839	0.861936	0.925293	0.03935	0.068593	0.068593	0.046656	0.02773
RDF	JACCARD	0.988208	0.935333	0.935333	0.940064	0.968732	0.012189	0.045923	0.045923	0.041486	0.021883
RDF	LPT	0.988208	0.877	0.877	0.901519	0.945486	0.012189	0.147801	0.147801	0.105277	0.061522
RDF	MAX_TSS	0.988208	0.935333	0.935333	0.940064	0.968732	0.012189	0.045923	0.045923	0.041486	0.021883
WMEA	JACCARD	0.986717	0.943667	0.943667	0.946368	0.972058	0.019012	0.045467	0.045467	0.042119	0.022325
WMEA	LPT	0.986717	0.878	0.878	0.897546	0.944354	0.019012	0.104976	0.104976	0.084042	0.04668
WMEA	MAX_TSS	0.986717	0.943667	0.943667	0.946368	0.972058	0.019012	0.045467	0.045467	0.042119	0.022325

Dataset S1 (separate file). Municipalities and relative proportion of savanna core areas across Brazilian three-banded armadillo distribution.

SI References

Bocchiglieri A, Mendonça AF, Henriques RPB (2012) Densidade e atividade do tatu-bola *Tolypeutes tricinctus* (Cingulata) em uma paisagem alterada no Brasil Central. In A Mastozoologia e a crise de Biodiversidade 6 Congresso Brasileiro de Mastozoologia. Corumb, Mato Grosso do Sul, 102-104.

Feijó A, Garbino GS, Campos BA, Rocha PA, Ferrari SF, Langguth A (2015) Distribution of *Tolypeutes* Illiger, 1811 (Xenarthra: Cingulata) with comments on its biogeography and conservation. Zoological Science **32**, 77-87.

Freitas MA, Silva TFS (2005) Guia Ilustrado dos Mamiferos na Bahia: especies continentais. Editora Uesb, Vitoria da Conquista, Brazil.

Hannibal W, Zortéa M, Calaça AM, Carmignotto AP, Bezerra AMR, Carvalho HG, Bonvicino CR, Martins ACM, Aguiar LMS, De Souza MB, Mattos I, Oliveira RF, Brito D, Silva DA, Guimaraes MA, Do Carmo EMB, Moreira JC (2021) Checklist of mammals from Goiás, central Brazil. Biota Neotropica **21,** e20201173.

Phillips SJ, Anderson RP, Schapire RE (2006) Maximum entropy modeling of species geographic distributions. Ecological Modelling **190**, 231–259.

Santos PM et al. (2019) NEOTROPICAL XENARTHRANS: a data set of occurrence of xenarthran species in the Neotropics. Ecology **100**, e02663.

Schetino MAA, Campos DP, Anacleto TCS, Santos FR (2021) New records of Tolypeutes tricinctus Linnaeus, 1758 (Cingulata: Tolypeutinae) in northern Minas Gerais state, Brazil. Lundiana **14**,1-4.

Nagy-Reis M et al. (2020) NEOTROPICAL CARNIVORES: a data set on carnivore distribution in the Neotropics. Ecology **101,** e03128.

Magalhães RA, Sena LMM, Rodrigues FHM (2021) First records of Brazilian three-banded armadillo (*Tolypeutes tricinctus*, Mammalia, Cingulata, Chlamyphoridae) predation by jaguar (Panthera onca, Mammalia, Carnivora, Felidae). Papeis Avulsos de Zoologia **61**, e20216158.
